# Investigation of risk factors for osteoporosis with a focus on hypertension and estimation of the causal effect of hypertension on osteoporosis using causal forest

**DOI:** 10.1038/s41440-025-02372-z

**Published:** 2025-10-02

**Authors:** Takuya Uematsu, Shuko Nojiri, Wataru Urasaki, Yuji Nishizaki

**Affiliations:** 1 Clinical Translational Science, Juntendo University School of Medicine Graduate School of Medicine, Tokyo, Japan; 2 Department of Hospital Pharmacy, Juntendo University Shizuoka Hospital, Shizuoka, Japan; 3 Medical Technology Innovation Center, Juntendo University, Tokyo, Japan; 4 Department of Information Science, Tokyo University of Science, Chiba, Japan; 5 Clinical Research and Trial Center, Juntendo University, Tokyo, Japan; 6 Division of Medical Education, Juntendo University School of Medicine, Tokyo, Japan

**Keywords:** Administrative claim database, Conditional average treatment effect, Hypertension, Osteoporosis, Risk

## Abstract

The current study aimed to comprehensively investigate the factors that most significantly increase the likelihood of developing osteoporosis, which is of great importance for aging populations. To this end, we focus on hypertension (HT) and examine its interaction and causal effect on osteoporosis. Using an administrative claims database, a nested case–control study and time-to-event analysis were conducted focusing on Japanese individuals aged ≥65 years. The results of the nested case−control study showed that rheumatoid arthritis (RA) had the highest odds ratio (OR = 1.961, 95% CI = 1.85–2.078), followed by HT (OR = 1.722, 95% CI = 1.659–1.787). In the time-to-event analysis, RA had the highest hazard ratio (HR = 2.133, 95% CI = 1.972–2.308), followed by chronic kidney disease (CKD) (HR = 1.473, 95% CI = 1.354–1.602), chronic obstructive pulmonary disease (HR = 1.46, 95% CI = 1.323–1.611), and HT (HR = 1.269, 95% CI = 1.21–1.331). Additionally, significant interactions were observed when HT co-existed with CKD, disorders of lipoprotein metabolism and other lipidemias (DLM), and RA. Moreover, the summary causal tree results of the conditional average treatment effect (CATE) using a causal inference approach revealed that the subgroup with DLM = 0, diabetes mellitus (DM) = 0, and RA = 0 exhibited the highest estimated CATE of 0.372, suggesting a strong independent causal effect of HT on osteoporosis in this group.

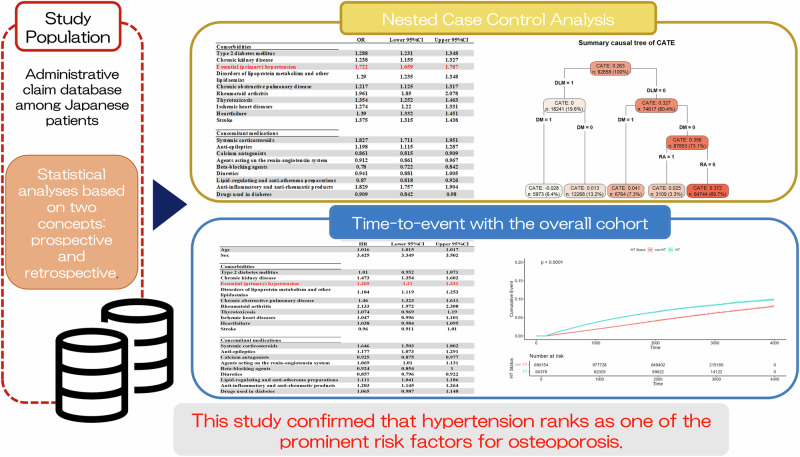

## Introduction

Osteoporosis is a skeletal disorder characterized by a reduction in bone density, which compromises bone strength and increases the risk of fractures. People with osteoporosis experience a myriad of challenges, including a decline in daily life activities and an increase in healthcare expenses. Estimates in Japan, which is a rapidly aging society, have shown that 12.8 million patients currently suffer from osteoporosis, constituting 10% of the total population (9.8 million women and 3 million men) [1]. Therefore, preventing osteoporosis, which is one of the underlying conditions for frailty, is crucial for extending the healthy life expectancy [2–4]. Osteoporosis can be classified into primary, triggered by aging and menopause, and secondary, induced by medications or diseases [5]. The mechanisms by which secondary osteoporosis develops vary depending on the causative disease. Some of the identified risk factors include inflammatory conditions such as rheumatoid arthritis (RA), endocrine disorders like thyrotoxicosis and diabetes mellitus (DM), and lifestyle-related diseases such as hypertension (HT) and chronic obstructive pulmonary disease (COPD) [6, 7].

Lifestyle-related diseases that increase with age often develop concomitantly with osteoporosis. However, evidence has shown that these conditions not only coexist but also significantly impact bone metabolism, including lifestyle-related diseases such as HT and COPD. Among them, HT has been estimated to affect approximately 43 million patients throughout Japan and serves as a risk factor for various diseases, including cerebrovascular disease [8]. The exorbitant costs of medications and treatments for HT and the resulting cerebrovascular disease have placed a significant burden on the healthcare economy of Japan [9]. Additionally, given that cerebrovascular disease is among the leading causes of mortality, comparable to cancer, effective blood pressure control is crucial [8, 10].

Investigating the correlation between HT, which has a prevalence of over 50% in both men and women aged 60 and above [8], and osteoporosis, the prevalence of which sharply increases in the 60s [1], can be considered clinically and economically meaningful for our rapidly aging populace. Recent reports suggest a potential shared pathological link between osteoporosis and HT, which has prompted numerous studies evaluating their association [11]. Although some studies have reported no association between the two conditions [12, 13], a majority indicate a significant correlation. To the best of our knowledge, no large-scale study has yet specifically and comprehensively investigated the association between osteoporosis and HT. Previous studies on osteoporosis have primarily relied on limited data and focused on specific factors, leading to inconsistent conclusions and dependence on limited evidence. In contrast, our study uses a large-scale dataset to comprehensively examine multiple risk factors, employing multiple statistical and study design methods, such as a nested case–control study and time-to-event analysis in the overall cohort, both of which consider temporal relationships. This multifaceted approach enhances the reliability of our findings. Research that derives results using such a comprehensive approach is likely to be pioneering in this field.

Furthermore, traditional analytical methods are considered inadequate for clarifying the causal relationship between HT and osteoporosis. However, causal inference approaches using real-world health data can provide evidence complementary to the results of randomized controlled trials [14]. In the case of observational data, the availability of evidence supporting heterogeneous treatment effects (HTEs) may offer an opportunity to personalize treatment decisions. In other words, the use of causal inference approaches allows for the estimation of treatment effects and the likelihood of developing certain diseases in different patient populations. The goal of predictive HTE analysis is to develop a model that can predict these effects while considering multiple variables, such as comorbidities that influence treatment outcomes and risks. Wager and Athey extended the random forest algorithm and developed a causal forest (CF) to estimate the HTE or conditional average treatment effect (CATE) within a causal inference framework for observational data. The CATE, which estimates the causal effects dependent on individual sample attributes rather than the entire population, has been used in causal inference that considers HTE. It is a useful estimator for evaluating individual characteristics with heterogeneous causal effects and for personalizing causal inference [15–17].

Based on the novelty of these approaches, the current study aimed to provide an overview of the predisposing factors that increase the likelihood of developing osteoporosis among the various diseases considered to be major risk factors for osteoporosis using a large-scale administrative claims database, with a particular focus on HT, and highlight signs warranting greater attention. While focusing on identifying risk factors, this study also examined the potential causal relationship between HT and osteoporosis and laid the foundation for exploring the mechanisms underlying this association in future research. To determine the causal relationship between HT and osteoporosis, we used a CF to estimate the CATE and identify the characteristics of individuals with heterogeneous causal effects.

Point of view

- Clinical relevance: Hypertension is an important risk factor for osteoporosis, alongside rheumatoid arthritis and chronic kidney disease. Particularly in older adults without comorbidities, hypertension has been shown to exert an independent causal effect on osteoporosis, making multifaceted preventive strategies centered on its management clinically important.
- Future direction: To further clarify the causal relationship between hypertension and osteoporosis, prospective studies incorporating factors such as disease severity, lifestyle, and inflammatory markers are essential. Moreover, it is essential to develop comprehensive risk assessment models that include these elements, as well as personalized models tailored to individual patient characteristics. Future research should also include interventional studies to evaluate the effects of antihypertensive treatment and longitudinal studies to examine the preventive impact of early hypertension management on osteoporosis.
- Consideration for the Asian population: In Asian populations, hypertension is highly prevalent, and salt intake tends to be higher than in other regions. Furthermore, distinctive patterns of bone growth and bone morphology (including bone area and shape), together with genetic factors specific to Asian populations, are reported to contribute to an increased risk of osteoporosis and fractures relative to other ethnic groups.

## Methods

### Data source and study cohort

This study utilized data collected retrospectively from the health insurance claims database provided by Japanese Medical Data Vision (MDV). This database covers approximately 22% of the Diagnosis Procedure Combination (DPC) hospitals, which are acute care hospitals widely distributed throughout Japan, with most DPC hospitals being acute care hospitals, and includes electronic health insurance claims data, DPC submission data, and laboratory examination data [18]. Furthermore, among the 35.23 million elderly individuals receiving medical care at 438 DPC hospitals under contract with MDV, approximately 1.16 million were randomly selected. The base cohort was registered in this database between April 2008 and December 2020, with the observation start and end dates for each patient being recorded.

Moreover, all previous diagnoses in the database are coded according to the International Statistical Classification of Diseases, 10th Revision (ICD-10). For medications, a unique nine-digit health insurance claims code is assigned to each drug based on therapeutic effects, which is then mapped to a five-digit code according to the Anatomical Therapeutic Chemical (ATC) Classification System managed by the European Pharmaceutical Marketing Research Association. Additionally, patient IDs and all personal information were anonymized.

Among the 1.16 million patients included in the database, data of patients aged ≥65 years who were first registered in this database between January 1, 2010, and December 31, 2015, were extracted, excluding those with <1 year of medical records. To set the window period, patients diagnosed with osteoporosis within 6 months of registration were excluded because such a diagnosis may have been established before their registration. The final population meeting these criteria was defined as the baseline cohort, which was treated as an open cohort. Follow-up was conducted until one of the following events occurred: osteoporosis diagnosis, censoring after loss to follow-up, or withdrawal of insurance coverage. All other patients were followed up until 2020. In this study, we performed statistical analyses based on two concepts: prospective and retrospective. For our retrospective analysis, the study participants were divided into those who were and were not diagnosed with osteoporosis. Thereafter, the presence or absence of HT was investigated using a nested case–control study. For our prospective analysis, the baseline study participants were divided into the HT and non-HT groups based on the presence or absence of comorbidities with HT. Incidence rates of new osteoporosis were then compared between these groups.

### Nested case–control study

The nested case–control study was performed as the main analysis. The primary weakness of conventional case–control study designs is their inability to verify whether cases and controls represent random samples from the same study base. However, nested case–control studies are theoretically “nested” within an explicitly defined cohort. This indicates that despite the difficulty of precisely defining the study base in traditional case–control studies, nested case–control studies have information available for all members of the cohort [19]. Consequently, this type of study provides multiple advantages, including reducing selection bias, clarifying chronological relationships, and allowing matching between cases and controls at specific time point (such as age) relative to outcome events. Moreover, such case–control studies use time point matching (risk-set sampling) within a clearly defined cohort wherein cases are those who had experienced the event of interest within the specified cohort, and controls are randomly sampled from the cohort at each respective time point [20]. After identifying the cases, those who did not develop osteoporosis at the time of matching were randomly selected as controls, with replacement from among cohort members who were at risk at the failure time of the case and matched at a 1:1 ratio based on age and sex.

Cases were defined by mapping the disease codes in the dataset to the diagnostic codes for osteoporosis in ICD-10 (M80 and M81), limited to confirmed diagnoses of the disease [21]. Patients newly diagnosed with osteoporosis during the study period were included in the osteoporosis cohort. Following the observation start date, the date at which each patient was newly diagnosed with osteoporosis was defined as the index date. Complications were defined as any disease diagnosed at least once prior to the index date. Similarly, concomitant medications were defined as any drug prescribed at least once within the 6 months prior to the index date.

According to a study by Yamana et al., the diagnoses in the DPC database showed a sensitivity of 78.9% and specificity of 93.2% [22].

### Time-to-event with the overall cohort

Taking into account the exclusion of study subjects through the matching process in the nested case–control study, a prospective time-to-event analysis was also conducted on the overall cohort as a secondary analysis. The index year was set to 2014, and patients were categorized into “patients with hypertension” and “patients without hypertension” based on the ICD-10 codes for hypertension. Complications, including HT, were defined as any disease diagnosed at least once within 6 months from the observation start date. Similarly, concomitant medications were defined as any drug prescribed at least once within this 6-month period.

### Assessment of risk factors

Medical risk factors for osteoporosis considered in this study were those identified from previous research as having a potential association with the onset of osteoporosis. These include the following conditions, with their corresponding ICD-10 codes being provided in Supplementary Table 1: type 2 DM, chronic kidney disease (CKD), essential (primary) HT, disorders of lipoprotein metabolism and other lipidemias (DLM), COPD, RA, thyrotoxicosis (TT), ischemic heart diseases (IHD), stroke, and heartfailure. Additionally, the following medications were included as confounding factors (Supplementary Table 2): systemic corticosteroids, anti-epileptics, calcium antagonists, agents acting on the renin-angiotensin system (RAS), beta blockers, diuretics, lipid-regulating and anti-atheroma preparations (lipid-lowering agents), anti-inflammatory and anti-rheumatic products (DMARDs), and antidiabetic drugs.

### Sensitivity analyses

In addition to the diagnostic code for osteoporosis, we included other drugs for disorders of the musculoskeletal system (ATC codes: M05B3, M05B4, M05B9, M05X0, A12A0, A11C2, G03J0, and H04E0) in the definition of osteoporosis and conducted a sensitivity analysis.

### Statistical analyses

Sex and the presence of complications were treated as binary variables. For age, sex, and comorbidity, the characteristics of cases and controls were defined using descriptive statistics and expressed as means, standard deviations, and proportions.

In the nested case–control study, the normal logistic model was extended to the corresponding binary data, and the conditional logistic regression model was applied to effectively improve the power by incorporating similarities between matched pairs. Odds ratios (ORs) along with their 95% confidence intervals (CIs) were estimated to determine the risk of osteoporosis incidence.

In the time-to-event analysis using the overall cohort, hazard ratios (HRs) were determined using an unmatched Cox proportional hazards model. This model determined person-years based on the time (in days) from the observation start date to the first diagnosis date, investigating the impact of HT on the incidence of osteoporosis. Patients who failed to develop osteoporosis by the end of the study period were censored. Kaplan–Meier survival curves were plotted to depict the cumulative incidence probability of osteoporosis, and differences between HT exposures were compared using the log-rank test. An exploratory analysis was conducted using a forest plot to visualize the impact of HT on osteoporosis within clinically relevant subgroups.

Furthermore, using data from the nested case–control analysis, the causal effect of HT on osteoporosis was adjusted for the following eight covariates that were specifically selected as high-risk factors for osteoporosis: DM, CKD, DLM, COPD, RA, TT, systemic corticosteroids, and anti-epileptics. We then evaluated the individual characteristics that show different causal effects and investigated those associated with higher causal effects by implementing a CF and examining the causal effect indicator, CATE. A causal forest was constructed using the R “grf” package. A summary causal tree was generated from the CF. The summary causal tree is a tool that randomly splits the target population and constructs a series of decision trees while evaluating how a random subset of predictor variables modifies the causal effect of hypertension on osteoporosis onset. The summary causal tree employed 2000 trees, with a minimum node size of 100 individuals for each tree. Each decision tree was constructed “honestly,” indicating that the entire dataset was randomly divided into 50% training data as the “split sample” and 50% test data as the “effect estimation sample” when developing the tree. The split sample was employed only for determining the tree’s branching structure, whereas the effect estimation sample was solely used for estimating the treatment effects at each terminal node. This separation keeps data used for splitting and estimation independent, thereby reducing bias in effect estimation[16, 23, 24]. The sample splitting ratio was specified using the honesty.fraction parameter; following standard practice, the data were equally split at a 50:50 ratio. This 50% split is recommended as it prevents overfitting and improves the precision and reliability of effect estimation by maintaining a balanced size between the splitting and estimation samples [25]. Within the effect estimation sample, cut-points for each variable were selected to maximize the difference in treatment effects between subgroups at the terminal nodes [26]. By adjusting the secondary sample, the decision trees focused on detecting meaningful heterogeneities in treatment effects. Furthermore, internal validation was performed using the tune.parameters option to optimize key parameters, including sample.fraction, mtry, and min.node.size, thereby enhancing treatment effect estimation accuracy. Internal validation mitigated the risk of overfitting and helped identify optimal parameter settings [23, 24, 26]. The model’s calibration was evaluated using the test_calibration() function from the “grf” package [24].

All analyses were conducted using R version 4.1.0 (http://www.r-project.org/). The “ccwc” package was used for nested case–control analysis, “grf” package for CF, and the “survival” package for the Cox proportional hazards model.

### Ethics statement

This study was conducted in compliance with the Ethical Guidelines for Medical and Biological Research Involving Human Subjects by the Ministry of Education, Culture, Sports, Science and Technology, and the Ministry of Health, Labour and Welfare, Japan. Our study was approved by the Ethics Committee for Medical Research of Juntendo University School of Medicine (Research permit number E21-0264-M01).

### Patient consent for publication

The Ethical Guidelines for Medical and Biological Research Involving Human Subjects by the Ministry of Education, Culture, Sports, Science and Technology and the Ministry of Health, Labour and Welfare, Japan waived the need for informed consent given that the study uses information that has already been anonymized (anonymized processed information).

## Results

### Characteristics of the study population in the nested case–control analysis

**Fig. 1:**
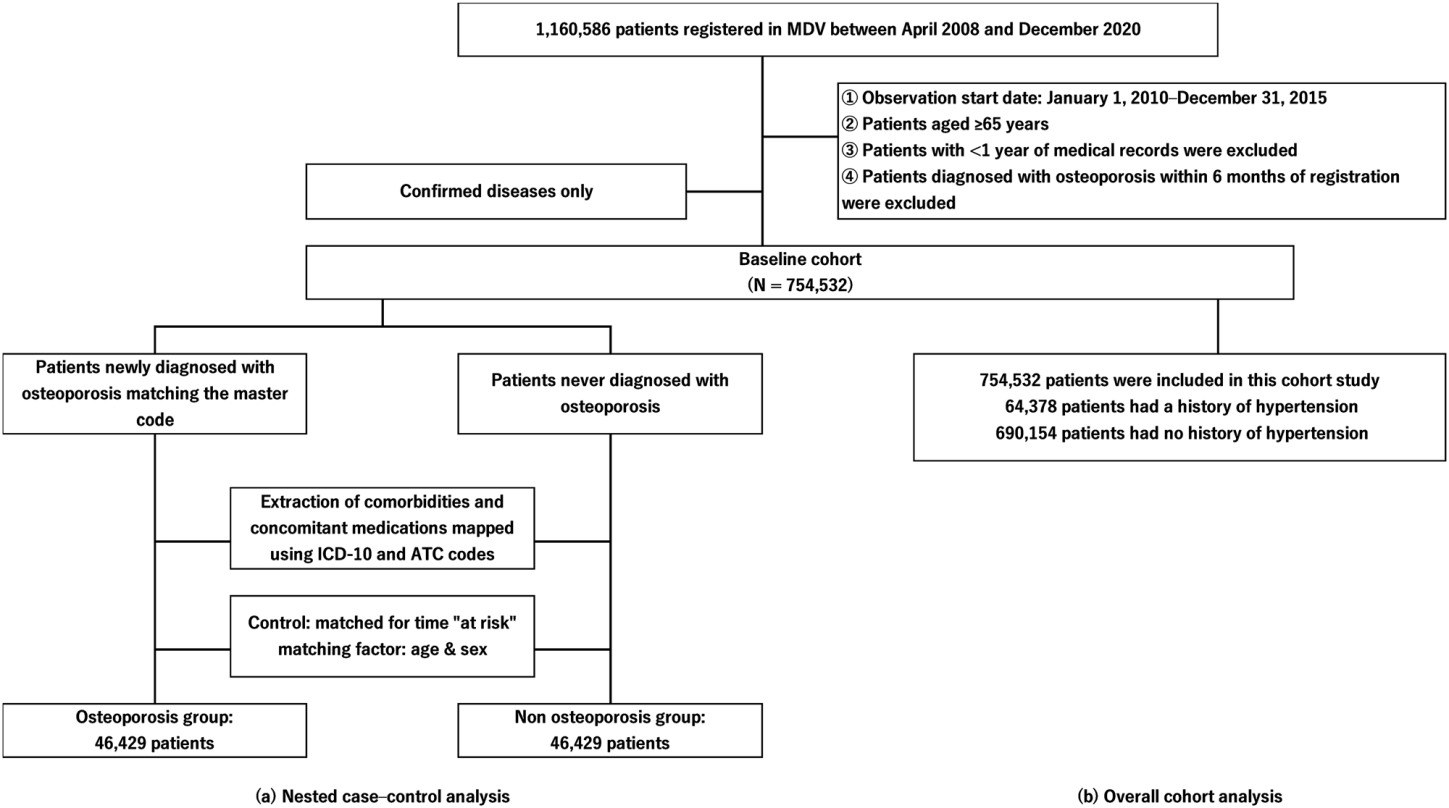
Flowchart showing the study design concept based on the MDV database. **a** Nested case–control analysis. **b** Overall cohort analysis. MDV, medical data vision

The baseline cohort comprised 754,532 individuals. After risk-set sampling with age and sex as matching factors, a final cohort of 46,429 patients with osteoporosis and 46,429 patients without osteoporosis (controls) was extracted (Fig. 1a). The average age at osteoporosis onset was 77.4 years (SD 7.5), with 21.4% and 78.6% male and female patients, respectively. The proportions of comorbidities and concomitant medications are summarized in Table 1(a).

**Table 1:** Characteristics of the study population in the nested case–control analysis and overall cohort analysis

	Nested case–control			Overall cohort		
	Osteoporosis group	Control group (non Osteoporosis group)	Std diff	Hypertension group	non Hypertension group	Std diff
	(n = 46429)	(n = 46429)		(n = 64378)	(n = 690154)	
Variable	N (%)	N (%)		N (%)	N (%)	
Age						
Mean (SD)	77.4 (7.5)	77.4 (7.5)	-	76.1 (7.2)	76.0 (7.7)	0.02
Sex						
Male	9952 (21.4)	9952 (21.4)	-	36325 (56.4)	322689 (46.8)	0.19
Female	36477 (78.6)	36477 (78.6)	-	28053 (43.6)	367465 (53.2)	
Complication						
Type 2 diabetes mellitus	9773 (21.0)	5279 (11.4)	0.26	14004 (21.8)	8178 (1.2)	0.68
Chronic kidney disease	3253 (7.0)	1675 (3.6)	0.15	4678 (7.3)	1714 (0.2)	0.38
Essential (primary) hypertension	22111(47.6)	12695 (27.3)	0.43	-	-	-
Disorders of lipoprotein metabolism and other lipidaemias	13639 (29.4)	7622 (16.4)	0.31	31250 (48.5)	11009 (1.6)	1.29
Chronic obstructive pulmonary disease	2414 (5.2)	1235 (2.7)	0.13	2808 (4.4)	3152 (0.5)	0.26
Rheumatoid arthritis	5814 (12.5)	2108 (4.5)	0.29	1670 (2.6)	2694 (0.4)	0.18
Thyrotoxicosis	2812 (6.1)	1162 (2.5)	0.18	2433 (3.8)	1871 (0.3)	0.25
Ischemic heart diseases	11058 (23.8)	5693 (12.3)	0.3	23414 (36.4)	11097 (1.6)	0.99
Heartfailure	12595 (27.1)	6370 (13.7)	0.34	21262 (33.0)	8999 (1.3)	0.93
Stroke	8214 (17.7)	4429 (9.5)	0.24	14112 (21.9)	10462 (1.5)	0.67
Concomitant medications						
Systemic corticosteroids	4750 (10.2)	1774 (3.8)	0.25	1890 (2.9)	2436 (0.4)	0.2
Anti-epileptics	2874 (6.2)	1535 (3.3)	0.14	2626 (4.1)	3453 (0.5)	0.24
Calcium antagonists	7967 (17.2)	4590 (9.9)	0.21	24549 (38.1)	1883 (0.3)	1.1
Agents acting on the renin-angiotensin system	7055 (15.2)	3991 (8.6)	0.2	27556 (42.8)	1045 (0.2)	1.22
Beta-blocking agents	2829 (6.1)	1692 (3.6)	0.11	9691 (15.1)	1299 (0.2)	0.58
Diuretics	4391 (9.5)	2356 (5.1)	0.17	9837 (15.3)	2237 (0.3)	0.58
Lipid-regulating and anti-atheroma preparations	6375 (13.7)	3605 (7.8)	0.19	19065 (29.6)	5458 (0.8)	0.88
Anti-inflammatory and anti-rheumatic products	11427 (24.6)	5698 (12.3)	0.32	7956 (12.4)	13819 (2.0)	0.41
Drugs used in diabetes	3336 (7.2)	1790 (3.9)	0.15	9109 (14.1)	3072 (0.4)	0.55
	(a) Baseline characteristics of patients with osteoporosis and controls in the nested case–control analysis			(b) Baseline characteristics of patients with and without hypertension in the overall cohort analysis		

*SD* standard deviation, *Std diff* standardized difference

### Nested case–control analysis

Table 2(a) shows the ORs and 95% CIs for univariate and multivariate analyses of other risk factors, including HT, for osteoporosis. Diseases with the highest risk of osteoporosis were RA (OR, 1.961; 95% CI, 1.85–2.078), followed by HT (OR, 1.722; 95% CI, 1.659–1.787).

**Table 2:** Multivariate analysis in the nested case–control analysis and overall cohort analysis

	Nested case–control						Overall cohort					
	Crude estimate			Adjusted estimate			Crude estimate			Adjusted estimate		
	OR	Lower 95%CI	Upper 95%CI	OR	Lower 95%CI	Upper 95%CI	HR	Lower 95%CI	Upper 95%CI	HR	Lower 95%CI	Upper 95%CI
Age	-	-	-	-	-	-	1.023	1.022	1.024	1.016	1.015	1.017
Sex	-	-	-	-	-	-	3.435	3.36	3.512	3.425	3.349	3.502
Comorbidities												
Type 2 diabetes mellitus	2.088	2.012	2.167	1.288	1.231	1.348	1.263	1.201	1.329	1.01	0.952	1.071
Chronic kidney disease	2.031	1.91	2.159	1.238	1.155	1.327	1.724	1.592	1.867	1.473	1.354	1.602
Essential (primary) hypertension	2.434	2.365	2.505	1.722	1.659	1.787	1.423	1.382	1.465	1.269	1.21	1.331
Disorders of lipoprotein metabolism and other lipidaemias	2.114	2.047	2.184	1.29	1.235	1.348	1.521	1.471	1.574	1.184	1.119	1.253
Chronic obstructive pulmonary disease	2.035	1.896	2.185	1.217	1.125	1.317	1.208	1.097	1.33	1.46	1.323	1.611
Rheumatoid arthritis	3.052	2.894	3.219	1.961	1.85	2.078	3.022	2.804	3.256	2.133	1.972	2.308
Thyrotoxicosis	2.517	2.346	2.7	1.354	1.252	1.463	1.575	1.425	1.741	1.074	0.969	1.19
Ischemic heart diseases	2.257	2.177	2.34	1.274	1.22	1.331	1.297	1.247	1.349	1.047	0.996	1.101
Heartfailure	2.361	2.28	2.444	1.39	1.332	1.451	1.372	1.316	1.43	1.038	0.984	1.095
Stroke	2.054	1.973	2.137	1.375	1.315	1.438	1.179	1.124	1.238	0.96	0.911	1.01
Concomitant medications												
Systemic corticosteroids	2.984	2.815	3.164	1.827	1.711	1.951	2.157	1.978	2.352	1.646	1.503	1.802
Anti-epileptics	1.936	1.817	2.064	1.198	1.115	1.287	1.384	1.264	1.515	1.177	1.073	1.291
Calcium antagonists	1.896	1.823	1.972	0.861	0.815	0.909	1.378	1.319	1.44	0.925	0.875	0.977
Agents acting on the renin-angiotensin system	1.92	1.842	2.003	0.912	0.861	0.967	1.431	1.373	1.492	1.069	1.01	1.131
Beta-blocking agents	1.721	1.618	1.832	0.78	0.722	0.842	1.257	1.172	1.348	0.924	0.854	1
Diuretics	1.973	1.872	2.08	0.941	0.881	1.005	1.353	1.268	1.443	0.857	0.796	0.922
Lipid-regulating and anti-atheroma preparations	1.891	1.81	1.975	0.87	0.818	0.926	1.589	1.523	1.659	1.111	1.041	1.186
Anti-inflammatory and anti-rheumatic products	2.326	2.244	2.41	1.829	1.757	1.904	1.47	1.403	1.54	1.203	1.145	1.264
Drugs used in diabetes	1.94	1.828	2.059	0.909	0.842	0.98	1.325	1.242	1.414	1.065	0.987	1.148
	(a) Crude and adjusted ORs and 95% CIs for osteoporosis in the nested case–control analysis						(b) Crude and adjusted HRs and 95% CIs for osteoporosis in the overall cohort analysis					

Odds ratios (ORs) and 95% confidence intervals (95% CIs) are estimated using conditional logistic models

OR, odd ratio; CI, confidence interval

Hazard ratios (HRs) and 95% confidence intervals (95% CIs) are estimated using Cox proportional hazard models

*HR* hazard ratio, *CI* confidence interval, *OR* odd ratio

### Characteristics of the study population in the overall cohort analysis

The study subjects were based on the baseline cohort of 754,532 individuals, ultimately including 64,378 patients with HT and 690,154 patients without HT (non-HT) (Fig. 1b). The HT group had an average age of 76.1 (SD 7.2) years, with 56.4% and 43.6% male and female patients, respectively. The non-HT group had an average age of 76.0 (SD 7.7) years, with 46.8% and 53.2% male and female patients, respectively. Table 1(b) summarizes the prevalence of comorbidities and concomitant medications.

### Time-to-event analysis

Table 2(b) shows the HRs and 95% CIs for univariate and multivariate analyses of other risk factors, including HT, for osteoporosis. The disease with the highest risk of osteoporosis was RA (HR, 2.133; 95% CI, 1.972–2.308), followed by CKD (HR, 1.473; 95% CI, 1.354–1.602), COPD (HR, 1.46; 95% CI, 1.323–1.611), and HT (HR, 1.269; 95% CI, 1.21–1.331).

**Fig. 2:**
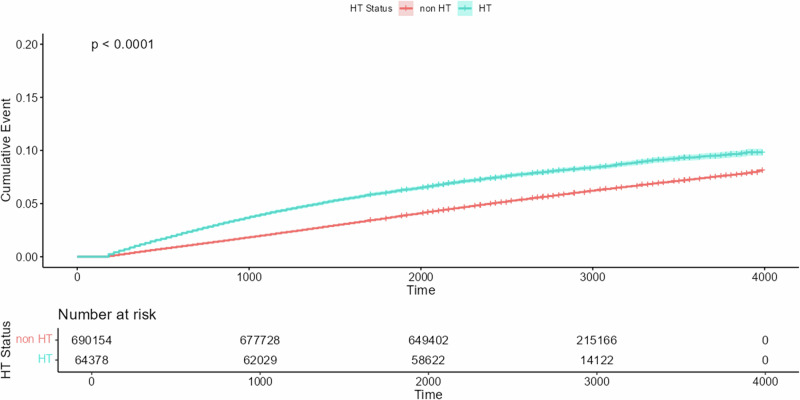
Kaplan–Meier curves showing osteoporosis risk in patients with hypertension. non-HT non-hypertension, HT Hypertension

Additionally, the relationship between osteoporosis and HT is depicted in Fig. 2 using Kaplan–Meier curves and the log-rank test. Our results demonstrated that the cumulative incidence rate of osteoporosis was significantly higher in the HT group than in the non-HT group (*p* < 0.001).

### Differences in osteoporosis risk across subgroups

**Fig. 3:**
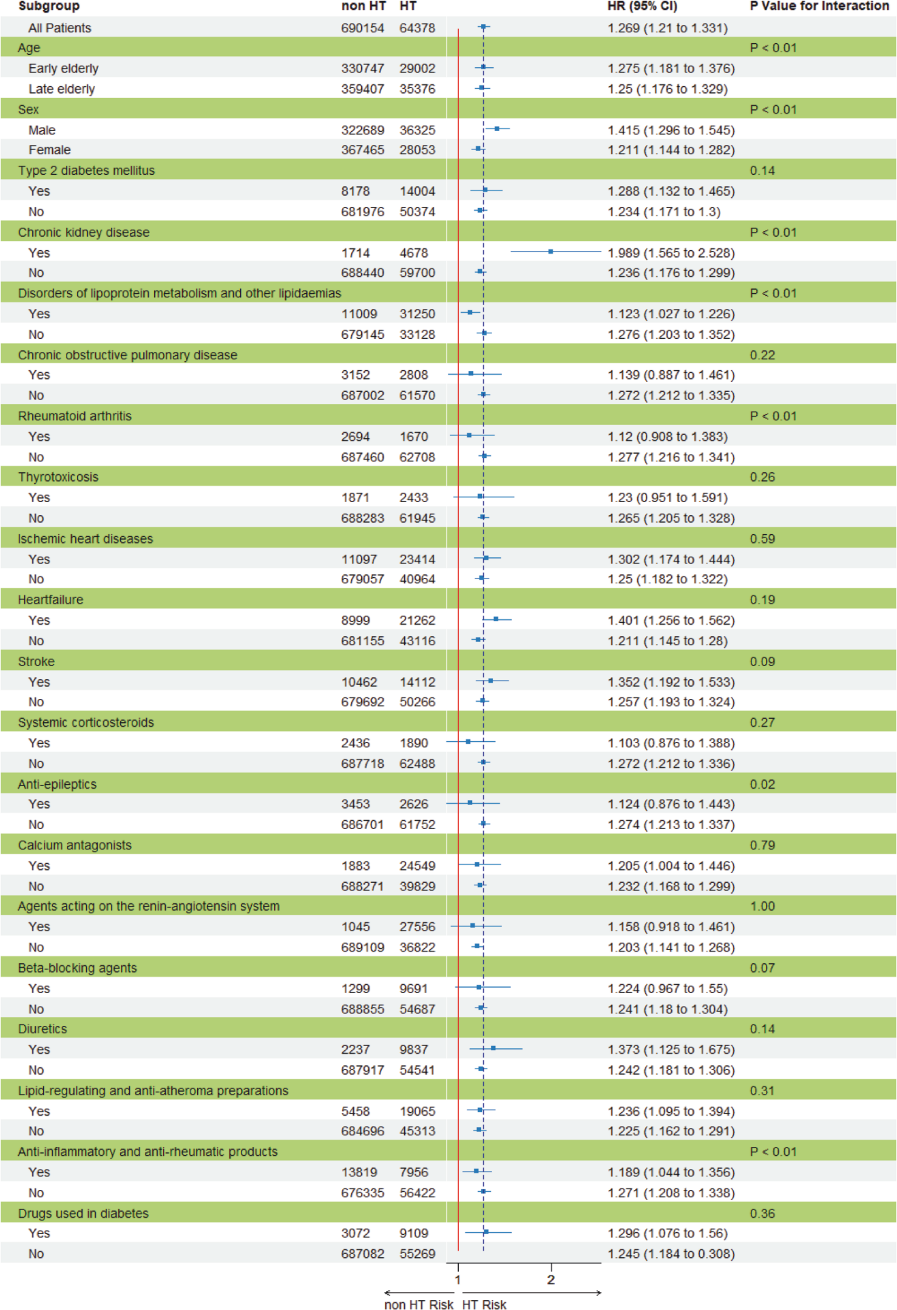
Hazard ratios for osteoporosis with the HT group versus the non-HT group, according to subgroup. non-HT non-hypertension, HT hypertension, early elderly 65–74 years, late elderly 75–100 years, HR hazard ratio, CI confidence interval

Figure 3 details the subgroup-specific HRs for osteoporosis between the HT and non-HT groups. Most subgroups showed similar HRs, with HT significantly increasing the risk of osteoporosis. Significant *p*-values for interaction were observed for CKD, DLM, and RA among the diseases and for anti-epileptics and DMARDs among the medications. Notably, the HR for HT decreased when combined with DLM or RA or when taking DMARDs. However, the HR for HT significantly increased when CKD was present.

### Sensitivity analysis

Our sensitivity analysis included medications for osteoporosis in the diagnostic codes. In the nested case–control study, each group comprised 28,135 individuals (Supplementary Table 3), with the highest risk observed for RA (OR, 2.088; 95% CI, 1.944–2.243), followed by HT (OR, 1.86; 95% CI, 1.774–1.95) (Supplementary Table 4). The overall cohort analysis included 62,343 patients with HT and 673,888 patients without HT (Supplementary Table 5), with the highest risk found for RA (HR, 2.472; 95% CI, 2.248–2.717), followed by COPD (HR, 1.588; 95% CI, 1.407–1.793), CKD (HR, 1.345; 95% CI, 1.203–1.505), and HT (HR, 1.291; 95% CI, 1.214–1.372) (Supplementary Table 6).

### CATE in the CF

**Fig. 4:**
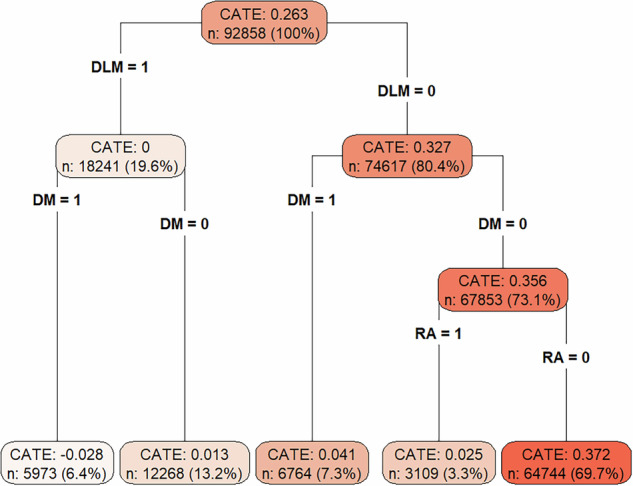
Summary causal tree of the conditional average treatment effect. Variables used in each node are presented, along with the estimated CATE, the number of participants (*n*), and the proportion (%) relative to the overall sample. CATE conditional average treatment effect, DLM disorders of lipoprotein metabolism and other lipidemias, RA rheumatoid arthritis, DM type 2 diabetes mellitus

The summary causal tree derived from the CF model, which was used for estimating the CATE of HT on osteoporosis risk, is presented in Fig. 4. Each node in the tree represents a subgroup defined by a combination of clinical covariates, with the estimated CATE shown accordingly.

In the subgroup with DLM = 0, DM = 0, and RA = 0, the estimated CATE was the highest at 0.372, indicating that HT significantly increases osteoporosis risk. This subgroup encompassed 64,744 individuals, accounting for 69.7% of the total sample. This finding suggests that, in a substantial portion of the population, the causal effect of HT on osteoporosis can be particularly strong. In contrast, in the subgroup with DLM = 1 and DM = 1, the CATE was the lowest at −0.028, suggesting a weak causal relationship between HT and osteoporosis in this group. This subgroup comprised 5973 individuals, representing 6.4% of the total sample.

Furthermore, the calibration evaluation of the model using test_calibration demonstrated that the mean and differential forest prediction coefficients were close to 1 (1.012 and 1.013, respectively) and statistically significant (both *p* < 0.01), indicating that the causal estimates from this model exhibited good calibration.

## Discussion

The significance of the current study lies in our demonstration of the relationship between osteoporosis and HT using real-world data, despite previous controversial reports, particularly in the context of Japan’s rapidly aging population. Furthermore, HT, along with RA, COPD, and CKD, was found to be one of the high-risk factors for osteoporosis. Furthermore, causal inference using the summary causal tree of the CATE revealed that patients without comorbid RA or DM demonstrated a markedly higher CATE, suggesting a stronger independent causal effect of HT on osteoporosis risk. This finding implies that HT can serve as a significant and independent etiological factor for bone fragility, irrespective of traditional high-risk comorbidities. These results emphasize the significance of integrating HT into comprehensive osteoporosis risk models, especially in populations that are inherently low risk. In terms of sex, women showed a higher risk for osteoporosis than did men, a finding consistent with the epidemiology observed in our country [1].

Bones continually undergo remodeling through the formation by osteoblasts and resorption by osteoclasts, maintaining bone mass through functional balance between these two distinct cell lineages. Osteoporosis develops when this regulatory mechanism fails and bone resorption relatively exceeds bone formation [27]. The mechanisms underlying the onset of this remodeling process imbalance vary depending on the underlying disease. In fact, guidelines from various countries, including Japan, cite endocrine disorders, lifestyle-related diseases, and RA as representative examples of causative diseases based on their clinical significance and frequency [1, 28–30].

HT, COPD, and DLM, which are chronic lifestyle-related diseases, have been identified as risk factors for osteoporosis that, unlike RA, do not directly affect the bones or joints. These conditions not only affect the respiratory and circulatory systems but also promote a decline in physical function, which is the greatest risk factor for osteoporosis, significantly impacting the patients’ daily lives. Therefore, lifestyle-related osteoporosis has been considered as a representative example of secondary osteoporosis.

Osteoporosis and HT, which have been referred to as silent diseases, are both lifestyle-related conditions that are rapidly increasing in countries facing super-aging populations, like Japan. Both conditions are influenced by both genetic and environmental factors, and their prevalence increases with age, among other common clinical characteristics. Although no previous studies have examined a sample size as large as that in the current study, they have demonstrated a relationship between osteoporosis and HT (Supplementary Table 7). However, the precise mechanism by which HT affects osteoporosis is still under debate and remains unclear, although several mechanisms have been reported. Recently, Pramusita et al. [31] who created a mouse model in which HT was induced by salt intake, discovered that osteoporosis was induced simultaneously with HT. In their attempts to elucidate the mechanism, they found that HT induction induced an increase in tumor necrosis factor-alpha (TNF-α) similar to that observed in RA, which promoted increased osteoclast formation by increasing the essential receptor activator of nuclear factor κB ligand (RANKL) for osteoclasts, thereby increasing bone resorption. Furthermore, they found that TNF-α increased the expression of angiotensin receptors in osteoblasts that express RANKL, and stimulation by angiotensin II increased the expression of RANKL from osteoblasts, thereby increasing osteoclast formation [31]. The most influential factor for bone metabolism is sodium intake. Excessive sodium intake increases sodium excretion in urine, consequently triggering increased excretion of calcium (Ca) in the urine. Hence, studies have reported that a decrease in serum calcium and an increase in parathyroid hormone promote bone resorption, ultimately causing bone resorption to surpass bone formation through accelerated bone metabolic turnover, resulting in a relative enhancement in bone absorption [32, 33].

The interaction between HT and CKD remains unclear, but the following mechanism is speculated. CKD is considered both a cause and a consequence of HT, with studies suggesting a strong interrelationship between CKD and HT [34]. Previous studies have shown that patients with CKD become highly sensitive to sodium due to the decline in renal function and that sodium intake is strongly correlated with the progression of CKD [35, 36]. Both CKD and HT are conditions characterized by an increase in sodium in the body, with their coexistence potentially promoting a significant increase in the HR for osteoporosis through the mechanism described earlier. Furthermore, the decrease in calcium concentrations due to disturbances in calcium balance and homeostasis in CKD patients is a physiological condition shared with those having osteoporosis, which may also contribute to the increase in HR [37].

On the other hand, RA is a systemic autoimmune disease primarily characterized by synovial joint inflammation, which causes the destruction of joint cartilage and bone [38, 39]. Additionally, systemic bone mineral density decreases, with osteoporosis being one of the major adverse outcomes of RA [40]. Numerous studies have focused on factors contributing to the ongoing loss of bone in RA. Notably, available evidence has shown that the expression of RANKL due to inflammatory cytokines, such as TNF-α, interleukin (IL)-1, and IL-6, and the concurrent use of glucocorticoids, causes an imbalance in the relationship between osteoblasts synthesizing the bone matrix and osteoclasts degrading bone tissue, leading to osteoporosis [41–43].

Regarding the interaction between HT and RA, the pharmacological effects of DMARDs, which are therapeutic agents for RA, has been considered significant. IL and TNF-α levels have been found to increase concurrently with the increase in blood pressure, inducing an increase in RANKL. However, these medications strongly inhibit inflammatory cytokines [44, 45]. Considering this opposite effect, we believe that HT complicated by RA may decrease the HR for osteoporosis.

The summary causal tree-derived results suggest that patients without comorbid DM or RA have a higher CATE of HT on osteoporosis risk than those with these comorbidities. This finding suggests that, in patients without DM or RA, the determinants of osteoporosis risk can be relatively limited and that HT can exert a strong independent causal effect on bone fragility. A potential explanation for this finding is that these patients have a lower baseline osteoporosis risk, making the relative impact of HT more evident, thereby causing a higher estimated CATE. In contrast, in patients with DM or RA, various pathophysiological processes, including chronic inflammation, abnormal glucose metabolism, or the use of corticosteroids and DMARDs, are already contributing to osteoporosis risk [46, 47]. Consequently, the added risk from HT may be relatively smaller.

Therefore, this tree-based analysis underscores that the impact of HT on osteoporosis risk significantly varies depending on the presence of comorbid conditions, providing significant implications for targeted risk assessment and the design of treatment strategies.

The current study has several limitations commonly associated with non-experimental database research. First, this study relied solely on a nested case–control study based on ICD-10 diagnostic codes, which may introduce biases due to coding errors or misclassifications. Second, each condition was diagnosed based on diagnostic codes recorded by physicians in health insurance claim data. Thus, some registration bias may potentially function as a confounding factor. Additionally, the absence of information regarding the severity of comorbidities and HT indicates that these factors were not considered in the analysis. Third, the data included in this study were limited to those from acute care hospitals in MDV, potentially representing patients with more severe conditions or multiple comorbidities rather than the general population. Additionally, given that diagnoses and prescriptions for each patient are recorded only within the same hospital, events diagnosed or treated outside the hospital, such as emergency admissions, may not have been included in the database. Fourthly, genetic factors, family history, and lifestyle factors were not considered in the study cohort, which may lead to residual confounding in the association between osteoporosis and HT.

Despite these limitations, this study has several strengths. First, the large sample size allowed for greater generalizability than conventional clinical trial data. By utilizing the nested case–control study design, definitions of exposure and covariates were both based on the results of risk-set sampling used in the selection of controls, enabling a temporally accurate evaluation of the relationship between osteoporosis and comorbidities. This allowed us to adjust for major confounders. Additionally, by carefully discussing the association between osteoporosis and HT using health administrative data, we provide robust evidence that would significantly benefit real-world clinical practice.

The results of this study emphasize that among the various risk factors for osteoporosis in elderly Japanese individuals based on real-world data, HT alongside RA, COPD, and DLM can be considered the most significant risk factors for osteoporosis. This finding highlights that HT alone demonstrates a strong causal effect on osteoporosis. Furthermore, evidence has shown that the risk of osteoporosis increases further when HT is accompanied by CKD. Patients with osteoporosis outnumber those undergoing pharmacological treatment for HT, and in today’s aging society, the management of lifestyle-related diseases, which rapidly increases with age, poses a significant social challenge. To prevent osteoporosis, individual risk factors and interactions that increase risk need to be considered in order to establish more comprehensive prevention strategies. We believe that this study will help address future global challenges, particularly in the effective prevention and treatment of osteoporosis.

### Asian perspectives

In Asia, the prevalence of hypertension has increased significantly in recent years, outpacing trends observed in many Western countries [48]. However, low awareness and treatment rates remain major public health challenges [49]. The findings of this study are significant for elderly healthcare in Asia, as they clarify the independent causal impact of hypertension—an often underrecognized factor—on osteoporosis. Given the regional disparities in healthcare resources and the high burden of multimorbidity among older adults in Asia, a multifaceted assessment of osteoporosis risk centered on hypertension management should be considered as part of future preventive healthcare and community-based integrated care strategies.

## Supplementary information

Supplementary Table 1

Supplementary Table 2

Supplementary Table 3

Supplementary Table 4

Supplementary Table 5

Supplementary Table 6

Supplementary Table 7

## Data Availability

Although the data are available from Medical Data Vision, the use of the data in this study is under license and not publicly available.

## References

[1] CftPaTo Osteoporosis. Osteoporosis Prevention and Treatment Guidelines. http://www.josteo.com/ja/guideline/doc/15_1.pdf. Accessed 17 Jan 2013.

[2] Chang CY, Tang CH, Chen KC, Huang KC, Huang KC. The mortality and direct medical costs of osteoporotic fractures among postmenopausal women in Taiwan. Osteoporos Int. 2016;27:665–76. 26243356 10.1007/s00198-015-3238-3

[3] Kobayakawa T, Miyazaki A, Saito M, Suzuki T, Takahashi J, Nakamura Y. Denosumab versus romosozumab for postmenopausal osteoporosis treatment. Sci Rep. 2021;11:11801. 34083636 10.1038/s41598-021-91248-6 PMC8175428

[4] Nishizawa Y, Nishizawa Y, Miura M, Ichimura S, Inaba M, Imanishi Y, et al. Executive summary of the japan osteoporosis society guide for the use of bone turnover markers in the diagnosis and treatment of osteoporosis. Clin Chim Acta. 2019;498:101–7. 31425674 10.1016/j.cca.2019.08.012

[5] El-Gazzar A, Högler W. Mechanisms of bone fragility: from osteogenesis imperfecta to secondary osteoporosis. Int J Mol Sci. 2021;22:625. 33435159 10.3390/ijms22020625 PMC7826666

[6] Gass M, Dawson-Hughes B. Preventing osteoporosis-related fractures: an overview. Am J Med. 2006;119:S3–11. 16563939 10.1016/j.amjmed.2005.12.017

[7] Kanis JA. Diagnosis of osteoporosis and assessment of fracture risk. Lancet. 2002;359:1929–36. 12057569 10.1016/S0140-6736(02)08761-5

[8] Guidelines for the Management of Hypertension (JSH 2019). https://www.jpnsh.jp/data/jsh2019/JSH2019_hp.pdf. Accessed 17 Jan 2023.

[9] Nakamura K, Okamura T, Miura K, Okayama A. Hypertension and medical expenditure in the Japanese population: review of prospective studies. World J Cardiol. 2014;6:531–8. 25068014 10.4330/wjc.v6.i7.531 PMC4110602

[10] Kabutoya T, Hoshide S, Kario K. Asian management of hypertension: current status, home blood pressure, and specific concerns in Japan. J Clin Hypertens. 2020;22:486–92. 10.1111/jch.13713 PMC8029793 31622008

[11] Hu X, Ma S, Yang C, Wang W, Chen L. Relationship between senile osteoporosis and cardiovascular and cerebrovascular diseases. Exp Ther Med. 2019;17:4417–20. 31105781 10.3892/etm.2019.7518 PMC6507516

[12] Hijazi N, Alourfi Z. Association between hypertension, antihypertensive drugs, and osteoporosis in postmenopausal Syrian women: a cross-sectional study. Adv Med. 2020;2020:7014212. 32149161 10.1155/2020/7014212 PMC7049845

[13] Javed F, Khan SA, Ayers EW, Aziz EF, Akram MS, Nadkarni GN, et al. Association of hypertension and bone mineral density in an elderly African American female population. J Natl Med Assoc. 2012;104:172–8. 22774384 10.1016/s0027-9684(15)30140-1

[14] Frakt AB. An observational study goes where randomized clinical trials have not. JAMA. 2015;313:1091–2. 25781429 10.1001/jama.2015.0544

[15] Wang T, Keil AP, Kim S, Wyss R, Htoo PT, Funk MJ, et al. Iterative causal forest: a novel algorithm for subgroup identification. Am J Epidemiol. 2024;193:764–76. 37943684 10.1093/aje/kwad219 PMC11485278

[16] Wager S, Athey S. Estimation and inference of heterogeneous treatment effects using random forests. J Am Stat Assoc. 2018;113:1228–42.

[17] Athey S, Tibshirani J, Wager. Generalized random forests. Ann Stat. 2019;47:1148–78.

[18] Vision MMD. https://www.mdv.co.jp/ebm/. Accessed Dec 2023.

[19] Essebag V, Genest J, Suissa S, Pilote L. The nested case-control study in cardiology. Am Heart J. 2003;146:581–90. 14564310 10.1016/S0002-8703(03)00512-X

[20] Langholz B, Goldstein L. Risk set sampling in epidemiologic cohort studies. Stat Sci. 1996;11:35–53.

[21] International Statistical Classification of Diseases and Related Health Problems. https://icd.who.int/browse10/2019/en. Accessed 5 Nov 2023.

[22] Yamana H, Moriwaki M, Horiguchi H, Kodan M, Fushimi K, Yasunaga H. Validity of diagnoses, procedures, and laboratory data in Japanese administrative data. J Epidemiol. 2017;27:476–82. 28142051 10.1016/j.je.2016.09.009 PMC5602797

[23] Edward JA, Josey K, Bahn G, Caplan L, Reusch JEB, Reaven P, et al. Heterogeneous treatment effects of intensive glycemic control on major adverse cardiovascular events in the ACCORD and VADT trials: a machine-learning analysis. Cardiovasc Diabetol. 2022;21:58. 35477454 10.1186/s12933-022-01496-7 PMC9047276

[24] Athey S, Wager S. Estimating treatment effects with causal forests: an application. Observational Stud. 2019;5:37–51.

[25] Lechner M, Okasa G. orf: ordered random forests. CRAN R package version 0.1, 2019; 3.

[26] Raghavan S, Josey K, Bahn G, Reda D, Basu S, Berkowitz SA, et al. Generalizability of heterogeneous treatment effects based on causal forests applied to two randomized clinical trials of intensive glycemic control. Ann Epidemiol. 2022;65:101–8. 34280545 10.1016/j.annepidem.2021.07.003 PMC8748294

[27] Noh JY, Yang Y, Jung H. Molecular mechanisms and emerging therapeutics for osteoporosis. Int J Mol Sci. 2020;21:7623. 33076329 10.3390/ijms21207623 PMC7589419

[28] Gregson CL, Armstrong DJ, Bowden J, Cooper C, Edwards J, Gittoes NJL, et al. UK clinical guideline for the prevention and treatment of osteoporosis. Arch Osteoporos. 2022;17:58. 35378630 10.1007/s11657-022-01061-5 PMC8979902

[29] Brown JP, Josse RG. Scientific Advisory Council of the Osteoporosis Society of Canada 2002 clinical practice guidelines for the diagnosis and management of osteoporosis in Canada. CMAJ. 2002;167:S1–34. 12427685 PMC134653

[30] Rossini M, Adami S, Bertoldo F, Diacinti D, Gatti D, Giannini S, et al. Guidelines for the diagnosis, prevention and management of osteoporosis. Reumatismo. 2016;68:1–39. 27339372 10.4081/reumatismo.2016.870

[31] Pramusita A, Kitaura H, Ohori F, Noguchi T, Marahleh A, Nara Y, et al. Salt-sensitive hypertension induces osteoclastogenesis and bone resorption via upregulation of angiotensin II Type 1 receptor expression in osteoblasts. Front Cell Dev Biol. 2022;10:816764. 35445013 10.3389/fcell.2022.816764 PMC9013777

[32] Cappuccio FP, Kalaitzidis R, Duneclift S, Eastwood JB. Unravelling the links between calcium excretion, salt intake, hypertension, kidney stones and bone metabolism. J Nephrol. 2000;13:169–77. 10928292

[33] Quereda C, Orte L, Sabater J, Navarro-Antolin J, Villafruela JJ, Ortuño J. Urinary calcium excretion in treated and untreated essential hypertension. J Am Soc Nephrol. 1996;7:1058–65. 8829122 10.1681/ASN.V771058

[34] Sinha AD, Agarwal R. Clinical Pharmacology of antihypertensive therapy for the treatment of hypertension in CKD. Clin J Am Soc Nephrol. 2019;14:757–64. 30425103 10.2215/CJN.04330418 PMC6500954

[35] de Brito-Ashurst I, Perry L, Sanders TA, Thomas JE, Dobbie H, Varagunam M, et al. The role of salt intake and salt sensitivity in the management of hypertension in South Asian people with chronic kidney disease: a randomised controlled trial. Heart. 2013;99:1256–60. 23766446 10.1136/heartjnl-2013-303688 PMC3756453

[36] McMahon EJ, Bauer JD, Hawley CM, Isbel NM, Stowasser M, Johnson DW, et al. A randomized trial of dietary sodium restriction in CKD. J Am Soc Nephrol. 2013;24:2096–103. 24204003 10.1681/ASN.2013030285 PMC3839553

[37] Moe SM. Calcium as a cardiovascular toxin in CKD-MBD. Bone. 2017;100:94–9. 27576942 10.1016/j.bone.2016.08.022 PMC5329167

[38] Hernández-Hernández V, Ferraz-Amaro I, Díaz-González F. Influence of disease activity on the physical activity of rheumatoid arthritis patients. Rheumatol. 2014;53:722–31. 10.1093/rheumatology/ket422 24369410

[39] Boers M, Kostense PJ, Verhoeven AC, van der Linden S, COBRA Trial Group. Combinatietherapie Bij Reumatoide Artritis Inflammation and damage in an individual joint predict further damage in that joint in patients with early rheumatoid arthritis. Arthritis Rheum. 2001;44:2242–46. 11665964 10.1002/1529-0131(200110)44:10<2242::aid-art386>3.0.co;2-f

[40] Chan MY, Center JR, Eisman JA, Nguyen TV. Bone mineral density and association of osteoarthritis with fracture risk. Osteoarthr Cartil. 2014;22:1251–58. 10.1016/j.joca.2014.07.004 25042553

[41] Nagayama Y, Ebina K, Tsuboi H, Hirao M, Hashimoto J, Yoshikawa H, et al. Low serum albumin concentration is associated with increased risk of osteoporosis in postmenopausal patients with rheumatoid arthritis. J Orthop Sci. 2022;27:1283–90. 34696921 10.1016/j.jos.2021.08.018

[42] Furuya T. Clinical observations of osteoporosis in Japanese patients with rheumatoid arthritis. Mod Rheumatol. 2022;32:839–45. 34979563 10.1093/mr/roab130

[43] Tella SH, Gallagher JC. Prevention and treatment of postmenopausal osteoporosis. J Steroid Biochem Mol Biol. 2014;142:155–70. 24176761 10.1016/j.jsbmb.2013.09.008 PMC4187361

[44] McInnes IB, Schett G. Pathogenetic insights from the treatment of rheumatoid arthritis. Lancet. 2017;389:2328–37. 28612747 10.1016/S0140-6736(17)31472-1

[45] Radu AF, Bungau SG. Management of rheumatoid arthritis: an overview. Cells. 2021;10:2857. 34831081 10.3390/cells10112857 PMC8616326

[46] Al-Hariri MT, Al Goweiz R, Aldhafery B, Alsadah MM, Alkathim AS, AlQassab MZ, et al. Potential cause affecting bone quality in Saudi Arabia: new insights. J Fam Med Prim Care. 2021;10:533–7. 10.4103/jfmpc.jfmpc_1872_20 PMC8132751 34017783

[47] Al-Hariri M. Sweet bones: the pathogenesis of bone alteration in diabetes. J Diabetes Res. 2016;2016:6969040. 27777961 10.1155/2016/6969040 PMC5061963

[48] Loo G, Puar T, Foo R, Ong TK, Wang TD, Nguyen QN, et al. Unique characteristics of Asians with hypertension: what is known and what can be done?. J Hypertens. 2024;42:1482–9. 38509747 10.1097/HJH.0000000000003706 PMC11296281

[49] Jin CN, Yu CM, Sun JP, Fang F, Wen YN, Liu M, et al. The healthcare burden of hypertension in Asia. Heart Asia. 2013;5:238–43. 27326143 10.1136/heartasia-2013-010408 PMC4832751

