# Supplementary material for: Investigation of risk factors for osteoporosis with a focus on hypertension and estimation of the causal effect of hypertension on osteoporosis using causal forest
Source: Hypertens Res. 2025 Oct 2;48(12):3067–79. doi: 10.1038/s41440-025-02372-z (PMC12678188; doi:10.1038/s41440-025-02372-z)
Supplement: Supplementary file 7 — Supplementary Table 7 [file 41440_2025_2372_MOESM7_ESM.docx]

**Supplementary Table 7. Summary of previous studies**

| First author |  | Year of  publication | Country | Study design | Study population | Statistical measurement of effect | Results |
| --- | --- | --- | --- | --- | --- | --- | --- |
| M.A. El-Heis  [1] |  | 2013 | Jordan | Cross-sectional study | 384 women | Prevalence of osteoporosis Women with hypertension = 20.3%  Women with no hypertension = 12.0%  p < 0.001 | Osteoporosis was significantly associated with current age, age at menarche, diabetes mellitus, hypertension, and renal problems. |
| S. Yang  [2] |  | 2014 | Australia | Longitudinal study | 2733 | Risk factor for any fragility fracture Hypertension: HR = 1.49  95% CI = 1.13–1.96 | Only women with hypertension had a lower femoral neck bone mineral density and higher risk of fracture than women without hypertension. |
| Mohammed Al-Hariri  [3] |  | 2020 | Saudi Arabia | Retrospective hospital-based analysis | 1332 | Correlation between hypertension and BMD T-score  r = −0.28  p < 0.001 | Pearson's correlation test revealed a significant negative correlation between hypertension and bone mineral density T-score. |
| Hao Chai  [4] |  | 2021 | China | Case–control study | 2039  natural postmenopausal women | Factors affecting osteoporosis Hypertension: OR = 1.303  95% CI = 1.012–1.677  p = 0.040 | Hypertension was significantly associated with osteoporosis. |
| Hai-Long Wu  [5] |  | 2022 | China | Cross-sectional study | 850 | Prevalence of osteoporosis  Hypertension group = 30.4%  Control group = 23.3%  p = 0.002 | The prevalence of osteoporosis was significantly higher among subjects with hypertension than among healthy controls. |
| Yuqing Huang  [6] |  | 2024 | US | Population-based cross-sectional study | 37807 (including 2523 osteoporosis patients) | Factors affecting osteoporosis  Hypertension: OR = 2.693  95% CI = 2.480–2.924  p < 0.001 | Hypertension was independently associated with osteoporosis in the general population. |

**Appendix References**

1. El Heis MA, Al Kamil EA, Kheirallah KA, Al Shatnawi TN, Gharaibia M, Al Mnayyis A. Factors associated with osteoporosis among a sample of Jordanian women referred for investigation for osteoporosis. East Mediterr. Health J 2013; 19: 459–64.

2. Yang S, Nguyen ND, Center JR, Eisman JA, Nguyen TV. Association between hypertension and fragility fracture: a longitudinal study. Osteoporos Int 2014; 25: 97–103.

3. Al-Hariri M, Aldhafery B. Association of hypertension and lipid profile with osteoporosis. Scientifica 2020; 2020: 7075815.

4. Chai H, Ge J, Li L, Li J, Ye Y. Hypertension is associated with osteoporosis: a case-control study in Chinese postmenopausal women. BMC Musculoskelet Disord 2021; 22: 1–7.

5. Wu HL, Yang J, Wei YC, Wang JY, Jia YY, Li L, et al. Analysis of the prevalence, risk factors, and clinical characteristics of osteoporosis in patients with essential hypertension. BMC Endocr Disord 2022; 22: 165.

6. Huang Y, Ye J. Association between hypertension and osteoporosis: a population-based cross-sectional study. BMC Musculoskelet Disord 2024; 25: 434.
